# Does High Voltage Electrical Discharge Treatment Induce Changes in Tannin and Fiber Properties of Cocoa Shell?

**DOI:** 10.3390/foods9060810

**Published:** 2020-06-19

**Authors:** Veronika Barišić, Ivana Flanjak, Mirela Kopjar, Mirta Benšić, Antun Jozinović, Jurislav Babić, Drago Šubarić, Borislav Miličević, Kristina Doko, Midhat Jašić, Đurđica Ačkar

**Affiliations:** 1 Faculty of Food Technology Osijek, Josip Juraj Strossmayer University of Osijek, Franje Kuhača 18, 31000 Osijek, Croatia; veronika.barisic@ptfos.hr (V.B.); mirela.kopjar@ptfos.hr (M.K.); antun.jozinovic@ptfos.hr (A.J.); jurislav.babic@ptfos.hr (J.B.); drago.subaric@ptfos.hr (D.Š.); bmilicevic@ptfos.hr (B.M.); dackar@ptfos.hr (Đ.A.); 2 Department of Mathematics, Josip Juraj Strossmayer University of Osijek, Trg Ljudevita Gaja 6, 31000 Osijek, Croatia; mirta@mathos.hr; 3 Polytechnic in Požega, Vukovarska 17, 34000 Požega, Croatia; 4 Federal Agro Mediterranean Institute, Biskupa Čule 10, 88000 Mostar, Bosnia and Herzegovina; kristina.skender@gmail.com; 5 Faculty of Technology Tuzla, Univerzitetska 8, 75000 Tuzla, Bosnia and Herzegovina; jasic_midhat@yahoo.com

**Keywords:** cocoa shell, high voltage electrical discharge, tannin, dietary fiber, water binding capacity, grindability

## Abstract

Cocoa shell is a by-product of the chocolate industry that is rich in dietary fiber and bioactive components. In this research, the influence of high voltage electric discharge (HVED) treatment on chemical and physical characteristics of the cocoa shell, i.e., the effects of applied time and frequencies on grinding ability, water binding capacity (WBC), dietary fibers and tannin content was investigated. HVED had a significant influence on the chemical and physical properties of cocoa shell, all of which could be linked to changes in fiber properties. Along with the fiber content, grinding ability and water binding capacity were increased. These properties have already been linked to fiber content and soluble/insoluble fiber ratio. However, this research implies that change in fiber properties could be linked to tannin formation via complexation of other polyphenolic components. Additional research is needed to verify this effect and to establish mechanisms of tannin formation induced by HVED and its influence on fiber quantification.

## 1. Introduction

Cocoa shell is the major by-product of the cocoa processing industry. It is a part of the cocoa bean that is separated from cotyledon during pre-roasting or after the roasting of beans [1]. It has been reported that several tons of cocoa shell need to be disposed annually, which poses a large problem [2,3]. Cocoa beans are rich in bioactive compounds, which are stored in the cotyledon. During fermentation, these components diffuse into cocoa shell, which becomes rich in bioactive compounds [4]. In addition, cocoa shell is rich in dietary fiber, mainly consisting of cellulose, carbohydrates and pectic polysaccharides [2] and presents great material for use in food industry and enrichment of food poor in dietary fibers. In the last few years, cocoa shell has been used as a raw biomass material, feedstuff, adsorbent, soil conditioner, garden mulch or burnt for fuel [5,6].

High voltage electric discharge (HVED) is a non-thermal process that has been used for the treatment of waste products from the food industry in the last few years [7]. It is also used as an extraction method, because it can disrupt the cellular walls and increase the overall mass transfer of the cellular content [8]. HVED is an innovative technique that interjects energy directly in aqueous solution between electrodes that are submerged. Electric discharge in water consists of two phases: corona streamer discharge process and arc discharge process. For the streamer discharge process weak shock waves are characteristic, as well as small number of bubbles and active radicals. When transiting to arc discharge process, number of bubbles is increased, shock waves become stronger, turbulence and concentration of free radicals are increased [9]. These shock waves and explosions of cavitation bubbles can affect particle size by fragmentation of cell membranes [10]. Electric discharge directly in water leads to production of molecular oxygen and hydrogen, hydrogen peroxide, hydroxyl radicals and oxygen radical ion, all of which are very reactive species.

Since HVED can disrupt cellular walls, which are in cocoa shell predominantly composed of cellulose with lesser amounts of hemicellulose and pectin [3], the aim of this study was to evaluate HVED influence on cocoa shell dietary fiber content and properties related to it. For use of cocoa shell in food industry, dietary fiber content, grindability, water and oil binding capacity and content of bioactive components are very important.

## 2. Materials and Methods

### 2.1. Preparation of Cocoa Shell

Cocoa shell samples were obtained after roasting fermented cocoa beans (West Africa mix supplied by Huyser, Möller B.V., Edam, Holland) at 135 °C for 55 min in custom made roaster (Metal workshop “ILMA”, Požega, Croatia). After that, the cocoa shell was easily separated by hand from the cotyledon.

Untreated cocoa shell (UCS) sample was obtained by grinding cocoa shell attained after separation from the cotyledon. Control samples were obtained by mixing the unmilled cocoa shell in water for 15, 30 and 45 min at concentrations of 1.5% and 3.0%. After mixing, the shell was separated from water and dried in the laboratory oven (Memmert, UFE 500, Schwabach, Germany) at 40 °C. Dry samples were ground in the laboratory mill (IKA, M20, Staufen, Germany) (25 g for 2 min with cooling) to obtain a fine powder (composite sample obtained by repeated grinding) and as such were frozen and stored for analyses. The grinded untreated cocoa shell was also frozen and stored for analysis in the same way as a cocoa shell mixed in water.

### 2.2. HVED Treatment

High-voltage electrical discharge equipment which was described by Barišić et al. [11] includes a chamber connected to a high-voltage pulse generator of 30 kV (the device was custom made by Inganiare CPTS1, Osijek, Croatia for the Faculty of Food Technology Osijek). Treatment chamber contains a stainless steel cylindrical needle (diameter 2.5 mm), and the ground electrode in the form of a plate (diameter 45 mm). Mixing of samples is achieved by magnetic stirrer. The distance between the electrodes during all treatments was 2 cm. Electric field density was 15 kV/cm during all treatments. HVED energy input ranged between 13.11–79.80 kJ/kg.

Unmilled cocoa shell (same as control samples prepared in water) was treated in HVED device at concentrations of 1.5% (6 g in 400 mL of distilled water) and 3.0% (12 g in 400 mL of distilled water). The treatment time was 15, 30 and 45 min, and the used frequencies were 40 and 80 Hz. Each sample (HVED, control or untreated) was treated until 200 g of sample was gained which gave us uniform sample for all analyses. The cocoa shell treated with HVED was dried, grind and stored until analyses in the same way as the control samples. Control samples (mixed in water) and HVED treated samples were dried to a dry matter content of 86.00 ± 0.85%.

### 2.3. Tannin Content

#### 2.3.1. Extraction

Each sample was weighed (2 g) and extracted three times with 10 mL of *n*-hexane (Carlo Erba Reagents, Val de Reuil, France) to remove lipids. Samples were dried at air over night and extracted with 5 mL 70% methanol (J. T. Baker, Deventer, Netherland) in ultrasound bath. After that, samples were centrifuged for 10 min at 3000 rpm (Sigma 2-16, Sigma, Osterode, Germany). Supernatant was decanted in 10 mL volumetric flask. That procedure was repeated twice after which flask with supernatant was filled up with 70% methanol.

#### 2.3.2. Spectrophotometric Analysis

Tannin content was determined by method described by Amorim et al. [12]. Method is based on binding of tannins with casein. Calibration curve was created with the standard solutions of tannic acid (Sigma-Aldrich, St. Louis, USA) in the range of concentrations from 0.5 to 3 mg/mL (y = 0.9011x + 0.0095; *R^2^* = 0.9993). Total phenol content and residual phenol content (obtained after complexation of tannin and casein) were determined spectrophotometrically at 760 nm according to the method of Singleton et al. [13]. Tannin content in prepared extracts was calculated Equation (1) as the difference between total phenol content and residual phenol content. Results are presented as mg of tannic acid per g of defatted sample (mg TA/g) and as a percentage of tannin in total phenol content (%).

(1) $$\mathrm{Tan}\mathrm{nin}\;\left(\frac{\mathrm{mg}\;\mathrm{TA}}{g}\right)=\mathrm{total}\;\mathrm{phenol}\;\mathrm{content}-\mathrm{residual}\;\mathrm{phenol}\;\mathrm{content}$$

### 2.4. Determination of Dietary Fibers

Dietary fibers were determined according to gravimetric AOAC method 991.43 [14]. Samples were treated with thermostable α-amylase, protease and amyloglucosidase (Megazyme Total Dietary Fiber Assay Kit, Megazyme Ltd., Bray, Ireland). The share of insoluble dietary fibers (IDF, %) was determined gravimetrically after filtration, and soluble dietary fibers (SDF, %) were determined by precipitation from the obtained filtrate. After correction for undigested protein (Kjeldahl method) and ash (mineralization at 525 °C), total dietary fibers were calculated Equations (2) and (3) as a sum of IDF and SDF. The values were calculated on the dry matter of the sample.

(2) $$\mathrm{Total}\;\mathrm{Dietary}\;\mathrm{Fibre}\;\left(\%\right)=\frac{\frac{R_{1}+R_{2}}{2}-p-A-B}{\frac{m_{1+}m_{2}}{2}}\times 100$$

(3) $$B=\frac{{\mathrm{BR}}_{1}+{\mathrm{BR}}_{2}}{2}-\mathrm{BP}-\mathrm{BA}$$

where: R_1_ = residue weight 1 from m_1_; R_2_ = residue weight 2 from m_2_; m_1_ = sample weight 1; m_2_ = sample weight 2; A = ash weight from R_1_; P = protein weight from R_2_; B = blank; BR = blank residue; BP = blank protein from BR_1_; BA = blank ash from BR_2_.

### 2.5. Grindability of Cocoa Shell

The grindability of cocoa shell was determined by sieving the powdered cocoa shell samples on analytical sieve shaker (Retsch GmbH, AS200, Haan, Germany) and measurement of mass of obtained fractions. A total of 50 g of the sample was sieved through six sieves (50, 71, 100, 125, 200 and 315 µm) during 15 min. After weighing each fraction, results were expressed as percentages of cocoa shell mass that was weighted on each sieve (%).

### 2.6. Water Binding Capacity (WBC) and Oil Binding Capacity (OBC)

For determination of WBC standard AACC Method 88-04 [15], was used. To 2.5 g of cocoa shell sample 30 mL of water was added. These solutions were left to stand at room temperature with periodic mixing. After that, the samples were centrifuged at 3000 rpm for 15 min (Centra-MP4R, IEC, Mumbai, India). The supernatant was decanted, and the remaining residue was weighted. The analysis was performed in two repetitions. The results were calculated Equation (4) and were expressed as grams of H_2_O absorbed per gram of cocoa shell (g/g).

(4) $$\mathrm{WBC}\left(\frac{g}{g}\right)=\frac{\mathrm{gel}\;\mathrm{mass}}{\mathrm{dry}\;\mathrm{matter}\;\mathrm{mass}\;\mathrm{in}\;\mathrm{the}\;\mathrm{initial}\;\mathrm{sample}}$$

For determination of OBC same procedure was used. The only difference was that for OBC instead of water cold pressed rapeseed oil was used. Results were expressed as grams of oil absorbed per gram of cocoa shell (%) obtained by formula Equation (5):

(5) $$\mathrm{OBC}\left(\frac{g}{g}\right)=\frac{\mathrm{gel}\;\mathrm{mass}}{\mathrm{dry}\;\mathrm{matter}\;\mathrm{mass}\;\mathrm{in}\;\mathrm{the}\;\mathrm{initial}\;\mathrm{sample}}$$

### 2.7. Fourier Transform Infrared Spectroscopy with Attenuated Total Reflection (FTIR-ATR) Analysis

FTIR–ATR spectra were recorded with a Cary 630 spectrometer (Agilent, Santa Clara, CA, USA) in wavenumber range from 4000 to 650 cm^−1^. For each sample, 32 scans were recorded and averaged with a spectral resolution of 16 cm^−1^.

### 2.8. Statistical Analysis

Statistical analysis was conducted using Statistica^®^, Version 13.4.0.14 (1984–2018 TIBCO Software Inc, Palo Alto, CA, USA). To determine the statistically significant difference of treatment effects, main effects and factorial analysis of variance (ANOVA) were used. P-value that was considered significant was 0.05. In addition, Pearson’s correlation coefficients was determined (*p* < 0.05).

## 3. Results and Discussion

### 3.1. Tannin Content

**Figure 1:**
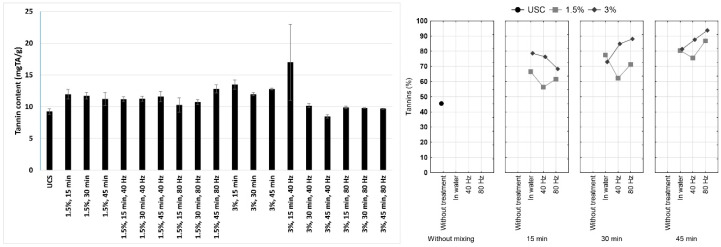
Tannin content (expressed on defatted sample weight) in cocoa shell before and after the high voltage electric discharge (HVED) treatment and percent of tannin in total phenols.

**Figure 2:**
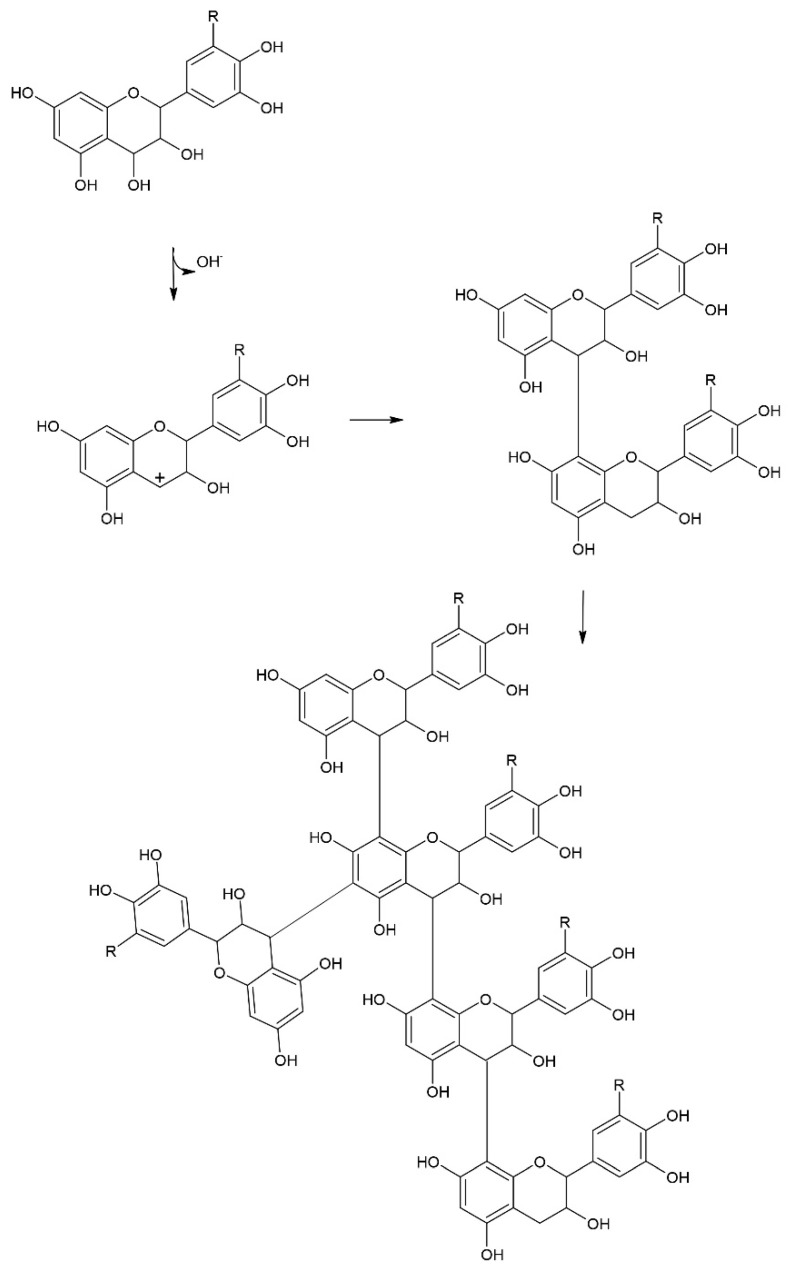
Condensation of polyphenols.

Tannin content of untreated cocoa shell and treated samples are shown in Figure 1 where results for tannin content (mg TA/g of defatted sample) and percentages of tannins in total phenols (%) are presented. It can be seen that the untreated shell had the lowest content of tannins, and the tannin content increased with all treatments. In samples treated with HVED, share of tannins in total phenols ranged from 45.03 to 65.09%. In our previous research [16], we have measured the decrease of content of all major polyphenolic compounds in cocoa shell treated with HVED (catechin, epicatechin, epicatechin gallate, gallic acid, caffeic acid and *p*-coumaric acid). These components are extractable by water, and the decrease in treated shell may have been the consequence of extraction, as reported by Jokić et al. [17], however, they are also prone to reactions of condensation in suitable conditions (Figure 2). Since the aim of this study was not to establish the effect of HVED treatment on extraction of bioactive compounds, cocoa shell was not milled before treatment, unlike in research of Jokić et al. [17]. Extraction yield is also dependent on electric field intensity, contact surface between material and solvent, liquid to solid ratio, etc. Considering above mentioned, HVED conditions applied in this research are not favorable for extraction [9]. Thus, extraction of polyphenolic compounds was aggravated. HVED generates different reactive species, which may have induced polymerization. Hence, HVED is a source of radicals that can easily oxidize tannins, which leads to an increase in their rigidity. Contrary to our results, Delsart et al. [18] and Lukić et al. [19] reported decrease of total tannin content in red wine treated by HVED and cold plasma, respectively, ascribing it to oxidation of tannins during treatment. However, one has to bear in mind that cold plasma and HVED treatment differ in that cold plasma includes gas introduction into liquid, and that there are major differences in chemical composition, mainly polyphenolic profile, of the treated samples. In addition, since treatment time in this research was significantly longer, oxidized tannins and other phenols might have been involved in mutual reactions, mainly because the oxidized phenols are hydrophobic. It has been reported that hydrophobic reactions can occur among polyphenols and induce their aggregation [20].

Results of tannin content, both in sample and in total phenols show that tannins are very resistant to HVED treatment. With the exception of 3.0% sample treated at 40 Hz, where significant reduction of tannin content occurred with the increase of treatment time, a slight increase of their content after treatment was observed (Figure 1), possibly due to oxidation and aggregation of tannins, but also due to the loss of a portion of soluble substances during treatment, which led to a change in the ratio of components in the samples.

Statistical analysis confirmed that tannins are very resistant to HVED treatment, since statistical significance was not established. Furthermore, factorial analysis of variance showed that influence of concentration and mixing time on percent of tannin in total phenols is statistically significant (Table 1). Coefficient of correlation is showing that tannin (%) is in relation with the smallest and largest particles, insoluble and total fibers. This may indicate that tannins have an impact on the proportion of fibers in cocoa shells, since the content of insoluble and total fibers increase as the proportion of tannin increases.

### 3.2. Dietary Fibers

**Figure 3:**
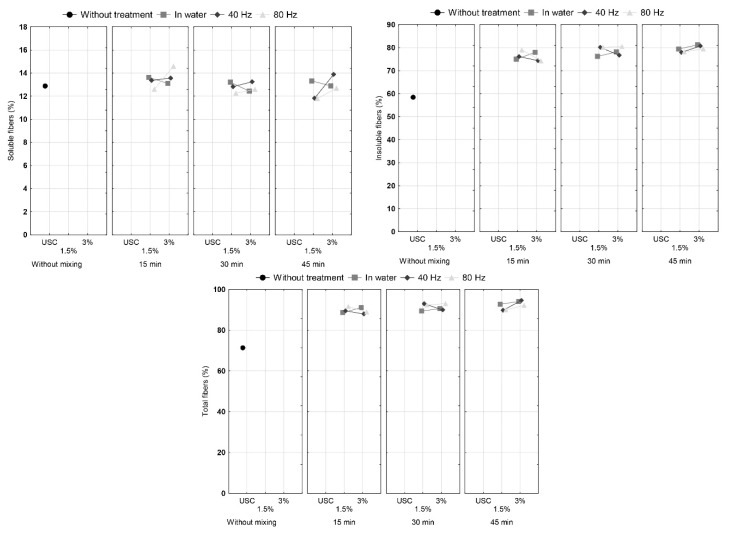
Content of insoluble, soluble and total fibers in cocoa shell before and after the HVED treatment.

Proportions of soluble, insoluble and total fibers of cocoa shell samples are shown in Figure 3. It can be seen that content of insoluble and total fibers in treated samples is higher than in untreated cocoa shell. The effect of HVED on soluble dietary fibers was not statistically significant (Table 2). An increase of insoluble fiber share after treatment had statistical significance for mixing time and there is a visible trend. A greater effect on increase of insoluble fiber content was in HVED treated samples at 1.5% concentration than at 3.0% due to greater energy input at lower sample concentration.

Increasing the fiber content in treated cocoa shells can be explained by the fact that during various treatments, fiber probably bonded with other components of the cocoa shell. However, some researches have shown that results obtained by gravimetric determination of fiber may be increased due to presence of insoluble proteins and condensed tannins [21,22,23]. The method used in this research has a step to exclude undigested proteins from the results for fiber content, however, condensed tannins may have an effect on the observed increase.

Condensed tannins are, along with resistant protein and Maillard reaction products, part of so-called Klason lignin [21], which is not always considered as a fiber. As shown by Perez et al. [24], roasted husk contains large amounts of free amino-acids and sugars, and our previous research [16] showed significant amounts of catechins. HVED generates free radicals and charged particles and highly reactive species (H^+^, OH^-^, H_2_O_2_), which may have induced advanced Maillard reactions and reactions of condensation of catechins to condensed tannins (Figure 2).

Our previous research showed that most likely 5-HMF and acrylamide are reacting with free radicals created by HVED and generating new compounds, which could be a part of Klason lignin [25]. This could contribute to increase of insoluble dietary fibers especially because condensed tannins and products of advanced Maillard reactions are insoluble in water.

In addition, we noticed that HVED treated samples had more undigested proteins than non-treated samples (results not shown). Decreased digestibility of proteins can be result of complex formation with tannins especially because HVED treatment generates change in pH and surface charge, which could be favorable conditions for complexation. Reduced in vitro and in vivo digestibility of proteins due to formation of complexes with tannins has already been reported for sorghum and several Acacia species. In addition, protein-protein complexation induced by tannins, and enzyme inhibition by tannins were also reported [26]. Although corrections for proteins were made, the other components that were bonded to them were not included here.

There are already some researches investigating the effect of electrical discharge on fibers. Yuan et al. [27] concluded that plasma improves the tensile strength and surface roughness, which leads to higher interfacial contact. In addition, during the treatment, it came to oxidation of fibers. Sinha and Panigrahi [28] observed increased hydrophobicity of jute fibers after plasma treatment, probably because of oxidation or decrease of phenolic and secondary alcoholic groups. Improved flexural strength of fibers occurred because of better adhesion between fibers and matrix. Bozaci et al. [29] and Karahan and Özdoğan [30] came to the conclusion that fibers have increased hydrophilicity, rougher surface and higher proportion of damaged fibers after plasma treatment.

Additional research is needed to reveal whether proposed mechanisms may be applicable to influence of HVED on fibers in cocoa shell.

### 3.3. Grindability of Cocoa Shell

The largest change in share of particles after HVED treatment was in the particle size ranges 0–50 μm and >315 μm (Table 3). Untreated cocoa shell had the largest percentage of particles between 0 and 50 μm and the smallest percentage of particles larger than 315 μm compared to treated and control samples. Any treatment, either only in water or with HVED, has led to an increase in the share of particles with size greater than 315 μm and a reduction in the share of particles with size less than 50 μm which was proven by coefficient of correlation (Table 4). There is a relation between the smallest and the largest particles. Main effect analysis of variance showed that there was a statistically significant difference between different sample concentrations during treatment for particle sizes of 0–50 μm, 51–71 μm and 72–100 μm (Table 2). In all treated samples decrease in the percentage of smaller particles and an increase in the percentage of larger particles was observed. The minimum change occurred in the sample 1.5%, 30 min, 40 Hz. Statistical analysis shows that there was a correlation between particle sizes and dietary fibers implying that difficulty to grind HVED treated cocoa shell can be caused by increased content of fibers.

### 3.4. Water and Oil Binding Capacity

**Figure 4:**
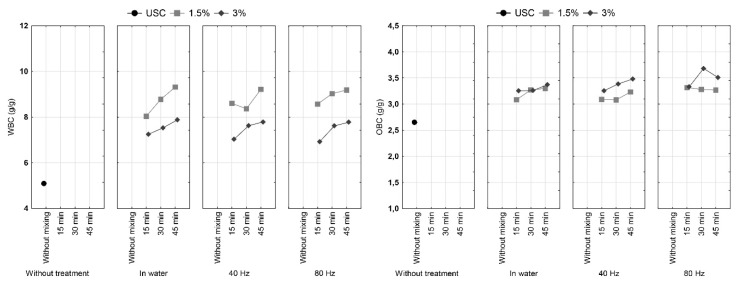
Oil and water binding capacity of cocoa shell before and after the HVED treatment.

WBC and OBC are important parameters for processing of food and any change in these properties influences production process. Water binding capacity (WBC) and oil binding capacity (OBC) of cocoa shell samples are shown in Figure 4. It is visible that the sample of untreated cocoa shell had the lowest WBC and OBC. Samples treated at a concentration of 1.5% had the higher WBC compared to samples treated at a concentration of 3.0%. OBC showed the opposite trend, where samples treated at 3.0% had higher capacity for binding oil than samples treated at 1.5%. The largest increases can be observed in samples treated for 45 min in water and with HVED. Statistical analysis showed that there was a statistically significant difference between tested concentrations and shearing time but treatment (with or without HVED) did not show statistical significance. All combinations of these effects have proven to be significant (Table 1).

According to Sangnark and Noomhorm [31], water and oil binding capacity are correlated to particle size. This research also revealed correlation of OBC with particle sizes (Table 4). Additionally, porosity, overall charge density and hydrophobic properties of fibers, all of which may be changed by HVED treatment, can greatly affect WBC and OBC [31,32]. This may also be substantiated by correlation of OBC with total fiber, insoluble fiber and tannin content in this research.

### 3.5. FTIR-ATR

**Figure 5:**
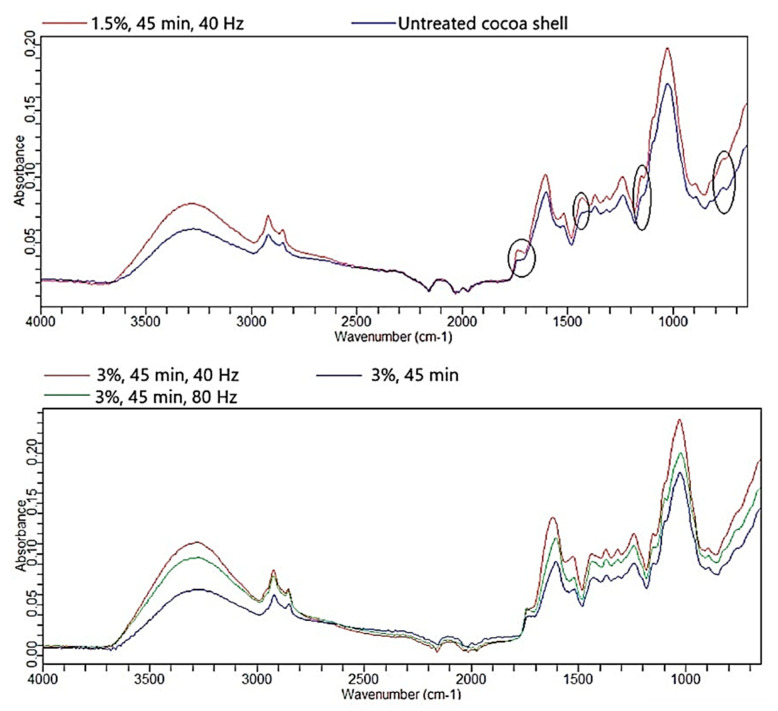
Representative Fourier transform infrared spectroscopy with attenuated total reflection (FTIR-ATR) spectra of cocoa shell before and after the HVED treatment.

The changes in chemical composition by HVED treatment were supported by FTIR-ATR analysis. All the treatments had similar trend so only representative spectra are shown in Figure 5.

In untreated cocoa shell C=O stretching at 1737 cm^−1^ is presented only with a shoulder, and there is a peak at 1602.8 cm^−1^. After the treatment, a small peak appears at 1737 cm^−1^. Karahan and Özdoğan [30] ascribed this peak to ester groups of pectin. This is implying that increased content of soluble fibers may be linked to the appearance of this peak after the treatment. However, according to Günzler and Gremlich [33] and Grillo et al. [34], this is also C=O stretch in unconjugated esters, carboxylic acids, aldehydes and ketones.

C-H asymmetric deformation vibrations in untreated shell are presented through a shoulder at 1410 cm^−1^, whereas after the treatments peak appears at 1431.3 cm^−1^.

In the untreated cocoa shell there is a peak at 1028.7 cm^−1^ with two shoulders at 1096 cm^−1^ and 1148 cm^−1^. Treatments did not change shoulder at 1096 cm^−1^, unlike the other one that has shifted to 1155 cm^−1^ (C-H deformation) and a small peak appears there. This is also close to peak (1152 cm^−1^) of C-O-C asymmetric vibration in carbohydrates and glucosides according to Grillo et al. [34].

Untreated cocoa shell had small peak at 760 cm^−1^ (ring deformation vibrations). Treatments transfers this to shoulder.

These changes in spectra are the result of combined effect of changes in fiber composition (insoluble:soluble ratio) and phenol changes. Bozaci et al. [29] also observed shift of the bands after cold plasma treatment of jute fibers. They assigned this to reaction of fibers with active species from the plasma.

## 4. Conclusions

In essence, our study showed influence of HVED on fiber properties (soluble, insoluble and total fiber content) and related physical properties—occurrence of larger particle size and increase of water and oil binding capacity. In addition, it has been established that changes in fiber properties correlate to changes in tannin content. It is evident that HVED has a significant influence on the physical and chemical characteristics of cocoa shells due to formation of large number of reactive species, including free radicals and ions, and the reactions occurring during treatment need to be further examined in order to see why such changes are taking place and to reveal actual mechanisms that are involved. Other chemical characteristics of the modified cocoa shell in future studies should be considered as well. In addition, the effect of change in physical and chemical properties of shell on its applicability in different foods needs to be examined because its properties such as grinding, taste, color etc. are the main reasons for its non-use in food production. This research is valuable for future applications of untreated and cocoa shells treated with HVED in the food industry.

## Figures and Tables

**Table 1:** Factorial analysis of variance.

		Sum of Squares	DF	Mean Square	F-Value	*p*-Value
OBC (g/g)	Intercept	392.4578	1	392.4578	533,103.9	<0.001 *
	Concentration (C)	0.2885	1	0.2885	391.8	<0.001 *
	Mixing (M)	0.1274	2	0.0637	86.5	<0.001 *
	Treatment (T)	0.1614	2	0.0807	109.6	<0.001 *
	C*M	0.0200	2	0.0100	13.6	<0.001 *
	C*T	0.0474	2	0.0237	32.2	<0.001 *
	M*T	0.0516	4	0.0129	17.5	<0.001 *
	C*M*T	0.0821	4	0.0205	27.9	<0.001 *
	Error	0.0133	18	0.0007		
WBC (g/g)	Intercept	2384.278	1	2384.278	313,752.4	<0.001 *
	Concentration (C)	15.149	1	15.149	1993.6	<0.001 *
	Mixing (M)	3.762	2	1.881	247.6	<0.001 *
	Treatment (T)	0.040	2	0.020	2.6	0.101638
	C*M	0.130	2	0.065	8.5	0.002470 *
	C*T	0.177	2	0.088	11.6	<0.001 *
	M*T	0.266	4	0.066	8.8	<0.001 *
	C*M*T	0.516	4	0.129	17.0	<0.001 *
	Error	0.137	18	0.008		
Tannin (mg TA/g of defatted sample)	Intercept	4714.410	1	4714.410	1021.198	<0.001 *
	Concentration (C)	0.030	1	0.030	0.006	0.936726
	Mixing (M)	13.343	2	6.671	1.445	0.261805
	Treatment (T)	17.066	2	8.533	1.848	0.186168
	C*M	24.240	2	12.120	2.625	0.099895
	C*T	10.946	2	5.473	1.186	0.328333
	M*T	29.441	4	7.360	1.594	0.218875
	C*M*T	24.668	4	6.167	1.336	0.294923
	Error	83.098	18	4.617		
Tannin (% of total polyphenols)	Intercept	114,239.9	1	114,239.9	3614.463	<0.001 *
	Concentration (C)	202.5	1	202.5	6.406	0.020914 *
	Mixing (M)	453.1	2	226.5	7.167	0.005134 *
	Treatment (T)	84.5	2	42.3	1.337	0.287391
	C*M	75.8	2	37.9	1.200	0.324306
	C*T	49.7	2	24.9	0.786	0.470508
	M*T	146.0	4	36.5	1.155	0.363192
	C*M*T	102.3	4	25.6	0.809	0.535434
	Error	568.9	18	31.6		

OBC: oil binding capacity; WBC: water binding capacity; DF: degree of freedom; * *p* < 0.05 statistically significant.

**Table 2:** Main effects analysis of variance.

	Effect	Sum of Squares	DF	Mean Square	F-Value	*p*-Value
0–50 µm	Intercept	169.1845	1	169.1845	59.27547	0.000006 *
	Concentration	20.7446	1	20.7446	7.26806	0.019455 *
	Mixing	2.4918	2	1.2459	0.43652	0.656146
	Treatment	2.8862	2	1.4431	0.50561	0.615433
	Error	34.2505	12	2.8542		
51–71 µm	Intercept	2025.733	1	2025.733	451.2232	<0.001 *
	Concentration	54.266	1	54.266	12.0875	0.004574 *
	Mixing	12.171	2	6.085	1.3555	0.294609
	Treatment	1.952	2	0.976	0.2174	0.807671
	Error	53.873	12	4.489		
72–100 µm	Intercept	1371.127	1	1371.127	705.1271	<0.001 *
	Concentration	18.601	1	18.601	9.5659	0.009312 *
	Mixing	0.303	2	0.152	0.0780	0.925440
	Treatment	1.729	2	0.864	0.4446	0.651247
	Error	23.334	12	1.945		
101–125 µm	Intercept	525.4466	1	525.4466	970.2258	<0.001 *
	Concentration	0.9016	1	0.9016	1.6648	0.221262
	Mixing	0.5083	2	0.2542	0.4693	0.636443
	Treatment	0.8918	2	0.4459	0.8234	0.462285
	Error	6.4989	12	0.5416		
126–200 µm	Intercept	3189.472	1	3189.472	1644.420	<0.001 *
	Concentration	1.189	1	1.189	0.613	0.448736
	Mixing	2.527	2	1.264	0.651	0.538775
	Treatment	2.636	2	1.318	0.679	0.525389
	Error	23.275	12	1.940		
201–315 µm	Intercept	5128.784	1	5128.784	3745.356	<0.001 *
	Concentration	0.147	1	0.147	0.107	0.748864
	Mixing	3.778	2	1.889	1.379	0.288933
	Treatment	4.432	2	2.216	1.618	0.238628
	Error	16.432	12	1.369		
>315 µm	Intercept	31,757.57	1	31,757.57	1505.873	<0.001 *
	Concentration	26.88	1	26.88	1.275	0.280959
	Mixing	45.68	2	22.84	1.083	0.369462
	Treatment	52.88	2	26.44	1.254	0.320301
	Error	253.07	12	21.09		
Insoluble fibers	Intercept	109,774.9	1	109,774.9	30,704.36	<0.001
	Concentration	0.1	1	0.1	0.02	0.883234
	Mixing	36.8	2	18.4	5.15	0.024326 *
	Treatment	2.7	2	1.4	0.38	0.691002
	Error	42.9	12	3.6		
Soluble fibers	Intercept	3038.191	1	3038.191	7378.913	<0.001 *
	Concentration	0.962	1	0.962	2.336	0.152303
	Mixing	2.116	2	1.058	2.570	0.117785
	Treatment	0.501	2	0.250	0.608	0.560488
	Error	4.941	12	0.412		
Total fibers	Intercept	149,338.0	1	149,338.0	43,452.47	<0.001 *
	Concentration	1.6	1	1.6	0.47	0.508137
	Mixing	21.7	2	10.9	3.16	0.079032
	Treatment	1.0	2	0.5	0.14	0.867489
	Error	41.2	12	3.4		

DF: degree of freedom; * *p* < 0.05 statistically significant.

**Table 3:** Grindability of cocoa shell samples before and after the HVED treatment.

Sample	0–50 µm (%)	51–71 µm (%)	72–100 µm (%)	101–125 µm (%)	126–200 µm (%)	201–315 µm (%)	>315 µm (%)
UCS	15.19	21.89	11.83	7.94	18.24	14.10	10.81
1.5%, 15 min	3.63	14.70	8.27	5.29	14.66	17.87	35.58
1.5%, 30 min	3.12	12.64	7.83	5.07	13.12	16.76	41.47
1.5%, 45 min	1.71	10.64	7.33	4.74	12.11	15.44	48.03
1.5%, 15 min, 40 Hz	5.64	13.78	7.42	5.52	13.62	17.42	36.61
1.5%, 30 min, 40 Hz	8.39	13.47	7.33	5.16	13.35	17.51	34.80
1.5%, 45 min, 40 Hz	3.67	13.67	7.42	5.15	12.65	16.94	40.50
1.5%, 15 min, 80 Hz	5.48	10.55	6.28	4.66	11.59	15.75	45.69
1.5%, 30 min, 80 Hz	2.64	11.71	8.59	5.50	13.33	16.75	41.48
1.5%, 45 min, 80 Hz	2.98	9.95	8.93	5.54	13.07	16.67	42.87
3.0%, 15 min	3.52	9.98	7.22	4.64	11.72	15.36	47.56
3.0%, 30 min	2.28	9.19	9.33	5.77	13.10	16.40	43.93
3.0%, 45 min	2.16	9.26	10.98	6.40	15.05	17.31	38.84
3.0%, 15 min, 40 Hz	1.36	8.89	11.45	5.79	15.12	18.76	38.64
3.0%, 30 min, 40 Hz	0.64	4.04	11.23	7.40	15.23	18.69	42.77
3.0%, 45 min, 40 Hz	2.08	8.84	10.14	5.24	12.78	16.18	44.75
3.0%, 15 min, 80 Hz	1.50	11.73	10.83	6.07	15.48	19.18	35.20
3.0%, 30 min, 80 Hz	1.32	6.51	9.05	4.92	12.47	15.72	50.01
3.0%, 45 min, 80 Hz	3.07	11.41	7.46	4.41	11.17	15.14	47.33

UCS: untreated cocoa shell.

**Table 4:** Pearson’s coefficients of correlation.

Variable	0–50 µm	51–71 µm	72–100 µm	101–125 µm	126–200 µm	201–315 µm	>315 µm	WBC (g/g)	OBC (g/g)	Insoluble Fibers (%)	Soluble Fibers (%)	Total Fibers (%)	Tannin (mg TA/g)	Tannin (%)
**0–50 µm**	1.000													
**51–71 µm**	0.839	1.000												
**72–100 µm**	0.006	−0.083	1.000											
**101–125 µm**	0.364	0.199	0.819	1.000										
**126–200 µm**	0.430	0.402	0.803	0.908	1.000									
**201–315 µm**	−0.456	−0.329	0.312	0.210	0.303	1.000								
**>315 µm**	−0.809	−0.795	−0.467	−0.717	−0.851	0.018	1.000							
**WBC (g/g)**	−0.252	−0.106	−0.473	−0.422	−0.391	0.096	0.349	1.000						
**OBC (g/g)**	−0.827	−0.838	−0.103	−0.479	−0.600	0.114	−0.862	0.233	1.000					
**Insoluble fibers (%)**	−0.765	−0.714	−0.455	−0.674	−0.751	0.264	0.883	0.450	0.776	1.000				
**Soluble fibers (%)**	−0.519	−0.351	0.098	−0.179	−0.023	0.582	0.268	0.306	0.334	0.334	1.000			
**Total fibers (%)**	−0.791	−0.723	−0.425	−0.666	−0.722	0.318	0.875	0.464	0.780	0.994	0.435	1.000		
**Tannin (mg TA/g)**	−0.244	−0.154	0.058	−0.089	0.035	0.357	0.097	−0.118	−0.100	0.123	0.065	0.127	1.000	
**Tannin (%)**	−0.768	−0.747	0.004	−0.365	−0.511	0.018	0.762	−0.014	0.844	0.635	0.167	0.627	0.067	1.000

Bold values were considered significant at *p* < 0.05.
